# Unraveling the Connection: Cholesterol, Calcium Signaling, and Neurodegeneration

**DOI:** 10.1177/26331055241252772

**Published:** 2024-05-11

**Authors:** Maria Casas, Eamonn J Dickson

**Affiliations:** 1 Department of Physiology and Membrane Biology, School of Medicine, University of California, Davis, CA, USA

**Keywords:** Calcium (Ca^2+^), Niemann-Pick type C1 (NPC1), membrane contact sites, neurodegeneration, voltage-gated calcium channel (Ca_V_), voltage-gated potassium channel

## Abstract

Cholesterol and calcium play crucial roles as integral structural components and functional signaling entities within the central nervous system. Disruption in cholesterol homeostasis has been linked to Alzheimer’s, Parkinson’s, and Huntington’s Disease while alterations in calcium signaling is hypothesized to be a key substrate for neurodegeneration across many disorders. Despite the importance of regulated cholesterol and calcium homeostasis for brain health there has been an absence of research investigating the interdependence of these signaling molecules and how they can tune each other’s abundance at membranes to influence membrane identity. Here, we discuss the role of cholesterol in shaping calcium dynamics in a neurodegenerative disorder that arises due to mutations in the lysosomal cholesterol transporter, Niemann Pick Type C1 (NPC1). We discuss the molecular mechanisms through which altered lysosomal cholesterol transport influences calcium signaling pathways through remodeling of ion channel distribution at organelle–organelle membrane contacts leading to neurodegeneration. This scientific inquiry not only sheds light on NPC disease but also holds implications for comprehending other cholesterol-associated neurodegenerative disorders.

## Introduction

Cholesterol and calcium play crucial signaling roles in the brain, influencing various cellular processes and functions. Cholesterol is a major structural component of neuronal cellular membranes where it influences rigidity and fluidity to impact and tune an array of neuronal events including synaptic formation,^1,2^ synaptic transmission,^3^ and ion channel function.^4^ Additionally, cholesterol is a key component of myelin, the fatty substance that wraps around nerve fibers, providing insulation and facilitating the rapid transmission of nerve impulses.^5-7^ Like cholesterol, calcium ions are crucial for neuronal signaling. They serve as second messengers in many intracellular signaling pathways with changes in intracellular calcium levels regulating processes such as neurotransmitter release, synaptic plasticity, and gene expression in neurons.^8-10^ Given the importance of both cholesterol and calcium for regulating neurological activity in health, it is perhaps not surprising that dysfunctional cholesterol^11,12^ and calcium levels^13-15^ contributes to various brain disorders.

This text discusses the role of cholesterol in the brain, particularly its importance in neuronal function and the impact of disruptions in cholesterol production or transport on the central nervous system and neurodegeneration. The narrative primarily focuses on the role of the Niemann-Pick type C (NPC) protein, specifically NPC1, in lysosomal cholesterol transport and provides insights and interpretations of published work^16-18^ investigating the molecular link(s) between loss of lysosomal NPC1 function, remodeling of membrane contact sites, and cytotoxic alterations in calcium signaling pathways leading to neurodegeneration. We discuss the molecular mechanisms governing cholesterol transport, and how alterations in intracellular cholesterol homeostasis initiate cellular programs that influence calcium signaling at organelle membrane contact sites to promote neurotoxicity. This work underscores the intimate relationship between cholesterol and calcium signaling, and highlights opportunities for potential therapeutic avenues.

## Cholesterol and Its Links to Neurodegeneration

Cholesterol is enriched in the brain and plays essential biophysical and signaling roles affecting ion permeability, cellular signaling, and transcription.^19^ Neurons obtain cholesterol from two sources: de novo synthesis in the Endoplasmic Reticulum (ER) and uptake of externally derived cholesterol-conjugated lipoproteins. Emerging evidence strongly supports a link between altered cholesterol homeostasis and neurodegeneration, with diseases such as Alzheimer’s disease (AD), Parkinson’s disease (PD), and NPC disease (NPC)^20^ reporting increases/accumulation of cholesterol and it’s metabolites, while Huntington’s disease (HD) studies have reported deficits in cholesterol metabolism that correlate with disease progression.^21,22^ Collectively, these reports emphasize that deviations from critical cholesterol setpoints may favor neurodegeneration. Supporting a role for elevated/accumulation of cholesterol in neurodegeneration, statins, a class of cholesterol-lowering drugs, have been investigated for their potential neuroprotective effects with studies in Alzheimer’s and NPC disease models demonstrating that statins reduce the risk of neurodegenerative diseases by modulating cholesterol metabolism and reducing neuroinflammation.^23-25^ However, the use of statins in neurodegenerative diseases remains a topic of debate, as conflicting results have been reported in clinical trials.^26,27^ Further, for neurodegenerative disorders that have deficits in cholesterol metabolism, statins may be expected to further reduce cholesterol levels and compound disease phenotypes. Thus, further research is needed to understanding the intricate mechanisms that link cholesterol to neurodegeneration to develop cholesterol-modifying therapeutic strategies to mitigate the progression of these devastating disorders.

**Figure 1.**
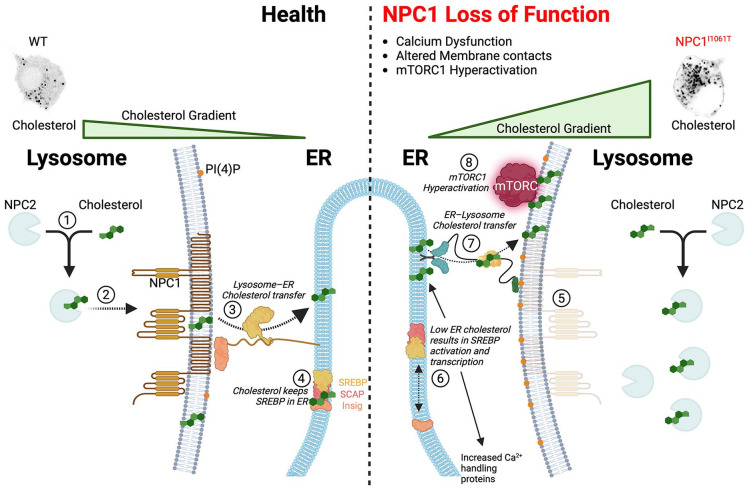
Role of NPC1 in cholesterol export from the lysosome. On the left, in a healthy neuron, the process of cholesterol transfer from external sources is outlined. Specifically, (1) lipoprotein-derived cholesterol binds to the NPC2 protein within the lysosome lumen, and (2) NPC2-bound cholesterol is then transferred to NPC1, facilitating the transport of cholesterol across the lysosomal membrane. (3) Further steps involve the transfer of cholesterol from the lysosome to the endoplasmic reticulum (ER) through sterol transfer proteins at lysosome-ER contact sites. (4) Cholesterol within the ER ensures the retention of the SREBP-SCAP-INSIG complex within the ER. On the right, the consequences of NPC1 loss of function or disease mutations are depicted. (5) NPC1 disease mutations lead to a decrease in the amount of functional NPC1 in lysosomal membranes, resulting in reduced cholesterol transport to the cytoplasmic side of the lysosomal membrane and the accumulation of cholesterol within the lysosome. (6) Disruption in cholesterol transport at ER-lysosome contacts leads to decreased ER cholesterol and the uncoupling of Insig from SREBP-SCAP. Consequently, the SREBP-SCAP complex is released from the ER, allowing it to be transported to the Golgi apparatus for proteolytic cleavage before being released to the nucleus, where it enhances the expression of cholesterol, phosphoinositide, and calcium handling proteins. (7) Increased production of PI(4)P^33^ on lysosome membranes recruits OSBP^32^ to ER– lysosome contacts for the transfer of cholesterol to lysosomal membranes. (8) Increased cholesterol on lysosomal membranes ultimately leads to the hyperactivation of mTORC1^32,40^.

The NPC proteins, specifically NPC1, play a crucial role in transporting cholesterol across the endo/lysosomal membrane prior to its transfer to the ER (Figure 1). Loss of function disease mutations in NPC1 lead to reduced cholesterol efflux from the lysosome and parallel cholesterol accumulation in the lysosome lumen (Figure 1, right) and cause NPC disease, a progressive neurodegenerative disorder with severe and early onset symptoms. Neurologically, altered cholesterol signaling in NPC patient neurons gives rise to impaired motor functions, dementia, and seizures. Despite clear neuropathological consequences for cholesterol dysregulation in NPC disease, the mechanisms through which cholesterol accumulation contributes to the neuropathology of NPC disease are poorly understood.

## NPC1 Cholesterol Transporter Influences Organelle Membrane Contacts

**Figure 2.**
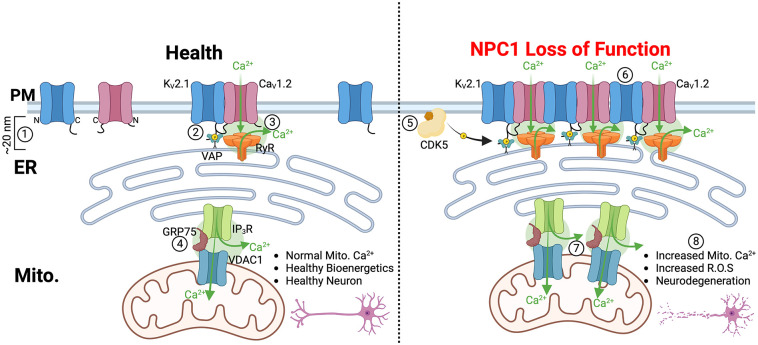
Loss of NPC1 function alters membrane contact sites to promote calcium cytotoxicity. On the left, in a healthy neuron, (1) Endoplasmic reticulum–plasma membrane contacts (ER–PM contacts) are regions where two organelle membranes come within approximately 20 nm of each other. (2) ER–PM contacts can be initiated by the phosphorylation of K_V_2.1, facilitating interactions with the ER protein, VAP. (3) Physical interactions among K_V_2.1, Ca_V_1.2, and RyR result in the creation of calcium nanodomains at ER–PM contacts. (4) Similarly, at ER–mitochondrial contacts, interactions between IP_3_R, GRP75, and VDAC1 lead to calcium transfer from the ER to mitochondria. On the right, following the loss of NPC1 function, (5) CDK5 drives phosphorylation of K_V_2.1, increasing its interactions with VAP and promoting enhanced clustering to Ca_V_1.2 and RyR. (6) This process results in elevated calcium entry into neurons. (7) Simultaneously, increased interactions between IP_3_R and VDAC1 promote excess calcium entry from the ER to mitochondria. (8) This leads to increased mitochondrial calcium, ultimately promoting neuronal death in vitro.

A key mechanism through which the lysosome communicates with other organelles is through physical membrane contacts. These intracellular synapses are intimate sites (~10-30 nm) of membrane contact between two or more organelles^28-31^ that are portals of information transfer akin to intracellular synapses (Figures 1 and 2). Unlike synapses, which use neurotransmitters, membrane contact sites use lipids and calcium to communicate information. A key signaling lipid that is transferred at endoplasmic reticulum (ER)– lysosome membrane contacts is cholesterol. NPC1-mediated cholesterol transport is a crucial rheostat that influences virtually every organelle through remodeling of membrane contact sites^17,18,32,33^ and hyperactivation of mTORC1 (Figure 1). Recent studies have demonstrated that NPC1 loss-of-function remodels ER-lysosome, ER-Golgi, and ER-mitochondria membrane contact sites (MCSs). However, there was an absence of information testing if it could also regulate ER–plasma membrane (PM) contacts. This is important because (i) 40 to 90% of cellular cholesterol is in the PM,^34,35^ therefore disruption of NPC1-mediated cholesterol would be expected to modify PM identity, and (ii) ER–PM contact sites are generated and regulated by phosphoinositides and Ca^2+^, two crucial signals that if grossly perturbed can cause neurodegeneration. One of the key regulators of somatic ER-PM MCSs in neurons is the voltage-gated potassium channel 2.1 (K_V_2.1). This channel not only regulates excitability but also generates ER-PM junctions through an association with ER-localized VAP proteins^36^ (Figure 2). K_V_2 channels also interact with Ca^2+^ signaling proteins like voltage-gated L-type Ca^2+^ channels (Ca_V_1), which are essential for neuronal excitability and gene expression.^37^ However, until recently there was a lack of evidence linking lysosomal cholesterol with regulation of ion channels at ER–PM contacts in neurons. In the study by Casas et al,^16^ disruption of NPC1 function or the presence of disease mutations was observed to elevate ER–PM MCSs, resulting in increased calcium entry (Figure 2, right). The underlying molecular mechanism driving this phenomenon involves CDK5-dependent phosphorylation of K_V_2.1. This phosphorylation event enhances K_V_2.1–VAP interactions, promoting a higher frequency of ER–PM membrane contacts. A secondary consequence of elevated K_V_2.1–membrane contact site formation is recruitment and clustering of voltage-gated L-type calcium 1.2 (Ca_V_1.2) channels at ER–PM contacts. Clustering of Ca_V_1.2 channels increases their opportunity for physical interactions with neighboring Ca_V_1.2 channels thereby enhancing the probability of cooperative gating^38,39^ leading to amplified calcium entry into neurons. Therefore, a cascade initiated by NPC1 loss of function reshapes the distribution and activity of Ca_V_1.2 channels at ER–PM calcium signaling nanodomains, leading to augmented calcium entry and elevated mitochondrial calcium levels, ultimately resulting in neuronal death.

## Calcium: Friend or Foe in NPC

The mechanism(s) leading to neurodegeneration in NPC disease is/are unknown. The positioning of lysosomes as crucial cellular signaling platforms suggests that more than one molecular mechanism may contribute to neuronal loss in NPC. Supporting complex disease pathogenesis, research has shown that various signaling pathways such as mTORC1,^40^ autophagy,^41^ mitochondrial dysfunction,^42^ and STING signaling^43^ are differentially regulated in NPC disease. Additionally, numerous calcium signaling pathways, spanning multiple organelles and membrane contact sites, are altered following NPC1 loss of function.^16-18,44-46^ Casas et al, adds to the list of reports documenting altered calcium signaling in NPC and details the molecular mechanisms linking enhanced neuronal ER–PM contact site formation to augmented calcium influx, elevated mitochondrial calcium levels, ultimately leading to neuronal death. Such a model supports the calcium hypothesis of neurodegeneration which posits that elevations in intracellular/organellar calcium are toxic for cells. However, such a model is likely an oversimplification and it is entirely possible that initial alterations in Ca^2+^ gradients across membrane contact sites represent cellular programs attempting to redistribute cholesterol rapidly and efficiently to cellular membranes to restore cholesterol homeostasis.^46-48^ Critically, unless new homeostatic cholesterol set points are found quickly, these gross alterations in Ca^2+^ gradients could provide a substrate for neurodegeneration.^17,18^ Supporting such a hypothesis, we and others have shown that targeting Ca^2+^ handling proteins can reduce cellular toxicity in models of NPC disease.^16,17,45^

## Treating the Origin or the Consequences of a Neurodegenerative Disorder

The most common NPC disease mutation is the substitution of an isoleucine for a threonine at the 1061 position (NPC1^I1061T^). This devastating amino acid exchange results in NPC1 misfolding and subsequent proteasomal degradation which results in approximately 90% functional reduction at the lysosomal limiting membrane. As with any genetic disorder debates center around the importance of treating the origin of the disease (i.e. increase the amount of functional NPC1 in the lysosomal membrane), the immediate consequence (i.e. cholesterol accumulation in the lysosome) or treating the downstream consequences of the mutation (i.e. target the signaling pathways responsible for toxicity). To increase the functional amount of NPC1 on lysosomal limiting membranes gene therapy remains an attractive option despite the regulatory issues and concerns about the ability to faithfully package and deliver this large protein to its desired locations. Other approaches to increase the functional amount of NPC1 include targeting its degradation and increasing the environment of the ER to enhance protein folding.^45^ There has been significant research attention around attempting to correct the cholesterol accumulation phenotypes in NPC disease using 2-Hydroxypropyl-β-cyclodextrin (i.e. HP-β-CD or VTS-270)^49,50^ to transport unesterified cholesterol across the lysosomal membrane into the cytosol thereby reducing its accumulation in the endo-lysosomes independently of NPC1 and NPC2 proteins. Casas et al demonstrate that the targeting CDK5 or K_V_2.1–Ca_V_1.2 interactions using pharmacological agents or synthetic peptides, respectively, holds the potential to successfully alleviate all subsequent cellular phenotypes. This includes the restoration of mitochondrial calcium levels and the mitigation of neurotoxicity observed in isolated neurons. These findings suggest a promising therapeutic avenue for addressing NPC1 disease. The hypothesis gains further support from data obtained in NPC animal models, revealing that targeting CDK5 has the capacity to impede disease progression.^51^ Additionally, insights from models of ischemic stroke and Alzheimer’s disease indicate that the targeting of K_V_2 channel clustering is effective in slowing down the progression of disease pathology.^52,53^ This collective evidence underscores the potential therapeutic significance of modulating CDK5 and/or K_V_2.1–Ca_V_1.2 interactions in addressing the cellular manifestations of neurodegeneration.

## Conclusion and Perspectives

In conclusion, the text presents findings that demonstrate the link between NPC1 function, cholesterol transport, and reorganization of calcium handling proteins at membrane contact sites. It emphasizes the role of K_V_2.1 and Ca_V_1 channels in this process and the potential for therapeutic interventions in NPC disease. The study suggests that disruptions in calcium signaling contribute to neurodegeneration in NPC disease and highlights the importance of understanding these mechanisms for potential treatments.
